# Impact of Elevated CO_2_ on Seed Quality of Soybean at the Fresh Edible and Mature Stages

**DOI:** 10.3389/fpls.2018.01413

**Published:** 2018-10-17

**Authors:** Yansheng Li, Zhenhua Yu, Jian Jin, Qiuying Zhang, Guanghua Wang, Changkai Liu, Junjiang Wu, Cheng Wang, Xiaobing Liu

**Affiliations:** ^1^Key Laboratory of Mollisols Agroecology, Northeast Institute of Geography and Agroecology, Chinese Academy of Sciences, Harbin, China; ^2^Centre for AgriBioscience, La Trobe University, Bundoora, VIC, Australia; ^3^Key Laboratory of Soybean Cultivation of Ministry of Agriculture, Soybean Research Institute, Heilongjiang Academy of Agricultural Sciences, Harbin, China; ^4^Beijing Key Laboratory of New Technology in Agricultural Application, College of Plant Science and Technology, Beijing University of Agriculture, Beijing, China

**Keywords:** soybean, climate change, mineral nutrients, protein, oil

## Abstract

Although the effect of elevated CO_2_ (eCO_2_) on soybean yield has been well documented, few studies have addressed seed quality, particularly at the fresh edible (R6) and mature stages (R8). Under the current global scenario of increasing CO_2_ levels, this potentially threatens the nutritional content and quality of food crops. Using four soybean cultivars, we assessed the effects of eCO_2_ on the concentrations of crude protein, crude oil, and isoflavones and analyzed the changes in free amino acids, fatty acids, and mineral elements in seeds. At R6, eCO_2_ had no influence on soybean seed protein and oil concentrations. At R8, eCO_2_ significantly decreased seed protein concentration but increased seed oil concentration; it also significantly decreased total free amino acid concentration. However, at the same stage, the proportion of oleic acid (18:1) among fatty acids increased in response to eCO_2_ in the cultivars of Zhongke-maodou 2 (ZK-2) and Zhongke-maodou 3 (ZK-3), and a similar trend was found for linoleic acid (18:2) in Zhongke-maodou 1 (ZK-1) and Hei-maodou (HD). Total isoflavone concentrations increased significantly at both the R6 and R8 stages in response to eCO_2_. Compared with ambient CO_2_, the concentrations of K, Ca, Mg, P, and S increased significantly under eCO_2_ at R6, while the Fe concentration decreased significantly. The response of Zn and Mn concentrations to eCO_2_ varied among cultivars. At R8 and under eCO_2_, Mg, S, and Ca concentrations increased significantly, while Zn and Fe concentrations decreased significantly. These findings suggest that eCO_2_ is likely to benefit from the accumulation of seed fat and isoflavone but not from that of protein. In this study, the response of seed mineral nutrients to eCO_2_ varied between cultivars.

## Introduction

Atmospheric CO_2_ concentration has risen from 280 ppm to 390 ppm in the last 250 years and is predicted to increase to 550 ppm by 2050 (Stocker et al., 2013). As the primary substrate for photosynthesis, elevated CO_2_ (eCO_2_) concentration in the atmosphere significantly influences plant growth and productivity in many crop species, especially in C3 crops where the CO_2_ saturation point is much higher than the current atmospheric CO_2_ levels (Ainsworth et al., 2007; Wang et al., 2008; Leakey et al., 2009). The positive effects of eCO_2_ on C3 crop yields have been documented in rice, wheat, cowpea, and soybean (Krishnan et al., 2007; Yang et al., 2007; Zhu et al., 2008; Bishop et al., 2015; Dey et al., 2017); however, relatively little research has focused on soybean seed quality, which is as important as yield (Thomas et al., 2003, 2009; Ziska et al., 2007).

Soybean [*Glycine max* (L.) Merr.] is the world’s most important legume and a major source of protein and oil, which contain essential free amino acids and fatty acids. Results from a previous study suggest that eCO_2_ has no effect on soybean seed protein concentration (Taub et al., 2008). This is perhaps because soybean crops alleviate nitrogen (N) deficiency by increasing N_2_ fixation under eCO_2_ and, thus, maintain seed N concentration with increased seed yield (Allen and Boote, 2000; Li et al., 2017). However, there are few studies in regard to the influence of eCO_2_ on amino acid concentration. On the other hand, several investigations show that eCO_2_ has either no effect (Thomas et al., 2003; Taub et al., 2008) or a positive effect on soybean oil concentration (Heagle et al., 1998; Hao et al., 2014). Heagle et al. (1998) reported that eCO_2_ significantly increases oleic acid concentration, whereas Thomas et al. (2003) found that fatty acid level shows no response to eCO_2_. Amid this controversy, the relevant underlying mechanisms warrant specific investigation.

The concentrations of several elements in seeds, such as iron (Fe), zinc (Zn), calcium (Ca), and magnesium (Mg), are significantly influenced by eCO_2_. For instance, Fe and Zn concentration in wheat, barley, and rice decrease under eCO_2_ (Fangmeier et al., 1997; Lieffering et al., 2004; Erbs et al., 2010). A significant decrease of 9.3% for Zn and 5.1% for Fe in wheat seed was reported in a meta-analysis of 64 relevant studies (Myers et al., 2014). In another study, a decrease in seed nitrogen (N), phosphorus (P), potassium (K), and sulfur (S) concentrations was also observed in barley (free air CO_2_ enrichment, FACE), potato (open-top chamber, OTC), wheat (FACE), and sorghum (OTC) (Prior et al., 2008; Högy and Fangmeier, 2009; Erbs et al., 2010). In soybeans, Hao et al. (2016) found a significant increase of 31% and 26% in seed P and K concentrations, respectively, under eCO_2_. However, in northeast China, which produces 33% of the nation’s soybean crop (Liu and Herbert, 2002), the effects of eCO_2_ on the concentrations of mineral elements in soybean seed have not been studied to date.

Soybean seeds contain large quantities of isoflavones, including daidzein, genistein, and glycitein, which are considered beneficial to human health (Bennett et al., 2004; Morrison et al., 2008; Medic et al., 2014). These chemicals inhibit ovarian and colon cancer cell growth (MacDonald et al., 2005; Chang et al., 2007) and lower serum low-density lipoprotein cholesterol levels (Taku et al., 2007). Whether eCO_2_ favors the accumulation of isoflavones in soybean seed remains largely unknown. Theoretically, the synthesis of isoflavones in the seed is closely associated with the availability of photosynthetic carbon, which generally increases under eCO_2_.

Moreover, vegetable soybean (edamame), collected at the immature stage (R6) before pods turn yellow, is very popular in East Asian countries and is becoming more popular in the United States and western countries. However, no study has focused on the influence of eCO_2_ on seed nutritional status at the fresh edible stage.

Using four soybean cultivars, we assessed the effects of eCO_2_ on the nutritional quality of soybean seeds at the fresh edible and mature stages. Specifically, we examined the concentrations of crude protein, oil, and isoflavones and analyzed the changes in free amino acids, fatty acids, and mineral elements in seeds. The present results offer valuable information to drive improvement in human nutrition under the rising global atmospheric CO_2_ scenario.

## Materials and Methods

### Research Site and Experimental Design

A pot experiment was conducted in OTC at the Northeast Institute of Geography and Agroecology (45°73’N, 126°61’E), Chinese Academy of Sciences, Harbin, China. The experiment had a random block design comprising two values for atmospheric CO_2_ concentration and four vegetable soybean cultivars with six replicates. The four vegetable soybean cultivars were Zhongke-maodou 1 (ZK-1), Zhongke-maodou 2 (ZK-2), Zhongke-maodou 3 (ZK-3), and Hei-maodou (HD). Before sowing, uniform seeds were selected and germinated on moistened filter paper at 25°C. After 2 days of germination, six seeds were sown in each pot containing 9 L of soil and subsequently thinned to two plants 10 days after emergence. Therefore, there were 12 pots per cultivar grown in either ambient CO_2_ (aCO_2_) or eCO_2_. Soil water content was maintained at 80 ± 5% of field capacity by weighing and watering. Three replicates were harvested at the R6 and R8 stages (Fehr et al., 1971). Seeds were then dried at 70°C for 72 h.

There were six octagonal OTCs with three for eCO_2_ and the remainder for aCO_2_. The OTCs had a steel frame; the main body was 2.0 m high with a 0.5 m high canopy, which formed a 45° angle with the plane (Zhang et al., 2014). The OTCs were covered with polyethylene film (transparency ≥95%). A similar OTC design has been widely used in other studies of CO_2_ (Liu et al., 2016; Yu et al., 2016; Chaturvedi et al., 2017). A digital CO_2_-regulating system (Beijing VK2010, China) was installed to monitor the CO_2_ levels in OTCs and to automatically regulate the supply of CO_2_ gas (99.9%) to achieve concentrations of 550 ± 30 ppm for eCO_2_ and 390 ± 30 ppm for aCO_2_.

### Chemical Analysis of Plant Samples

The Soxhlet extraction method was used to determine the total oil concentration in seeds. To achieve this, 0.5 g of dried sample was weighed and wrapped tightly using a weighted piece of filter paper and was placed into the Soxhlet apparatus in a water bath maintained at 60°C. Subsequently, 200 mL of ethyl ether was added to the Soxhlet apparatus to extract the oil. After a 48-h extraction period, the defatted sample was placed in an oven at 45°C for 12 h, and the weight was used to calculate the oil content (Li et al., 2014).

Crude protein concentration was determined using the combustion N analysis method by Elementar-Vario (Elementar Analysensysteme GmbH E-III, Germany). Total N was converted to crude protein content using a conversion factor of 6.25 (Saldivar et al., 2011). Free amino acid concentrations were determined by reverse-phase high-performance liquid chromatography (RP-HPLC), as described by Qin et al. (2014). The concentrations of 16 amino acids were measured and analyzed, these included the following: aspartate (Asp), threonine (Thr), serine (Ser), glutamate (Glu), proline (Pro), glycine (Gly), alanine (Ala), valine (Val), methionine (Met), isoleucine (Ile), leucine (Leu), tyrosine (Tyr), phenylalanine (Phe), histidine (His), lysine (Lys), and arginine (Arg).

The isoflavone concentration was determined by HPLC using the method described by Sun et al. (2011) with slight modifications. Dried seed samples (0.5 g) were placed in 10 mL of 70% methanol solution; after shaking for 8 h at 240 rpm, the mix was centrifuged at 4000 rpm for 10 min and then filtered through a 0.45 μm filter. A detailed method for the determination of isoflavone concentration is described by Wu et al. (2016).

Fatty acid concentration was determined by gas chromatography (GC) with a flame ionization detector. A total of 0.33 g of dried seed sample was placed in n-hexane solution for 5 h after a 0.5 min vortex. The supernatant was used for methyl esterification; later, the concentrations of the five fatty acids were determined according to the method described by Qin et al. (2014).

For seed digestion, 0.5 g of dried seed was placed in 10 mL of HNO_3_ and 2.5 mL of HClO_4_ acid (v/v 4:1) for 24 h at room temperature. Later, the seed samples were digested in the digestion instrument until clear liquid was obtained; subsequently, the liquid was diluted to 25 mL. The concentrations of Fe, Cu, Mg, Mn, and Zn were analyzed by ?ame atomic absorption spectrometry, and the concentrations of K and Ca were determined using a flame photometer. Each measurement was repeated five times.

### Data Analysis

The mean data were compared according to Duncan’s multiple range test at 5% significance. Two-way analysis of variance (ANOVA) on variables such as the chemical element concentration, protein concentration, oil concentration, fatty acid concentration, and isoflavone concentration was performed to assess the interaction between CO_2_ and cultivar at levels of significance of *P* = 0.05, *P* = 0.01, and *P* = 0.001, using Genstat 13 (VSN International, Hemel Hempstead, United Kingdom).

## Results

### Protein and Free Amino Acids

**FIGURE 1:**
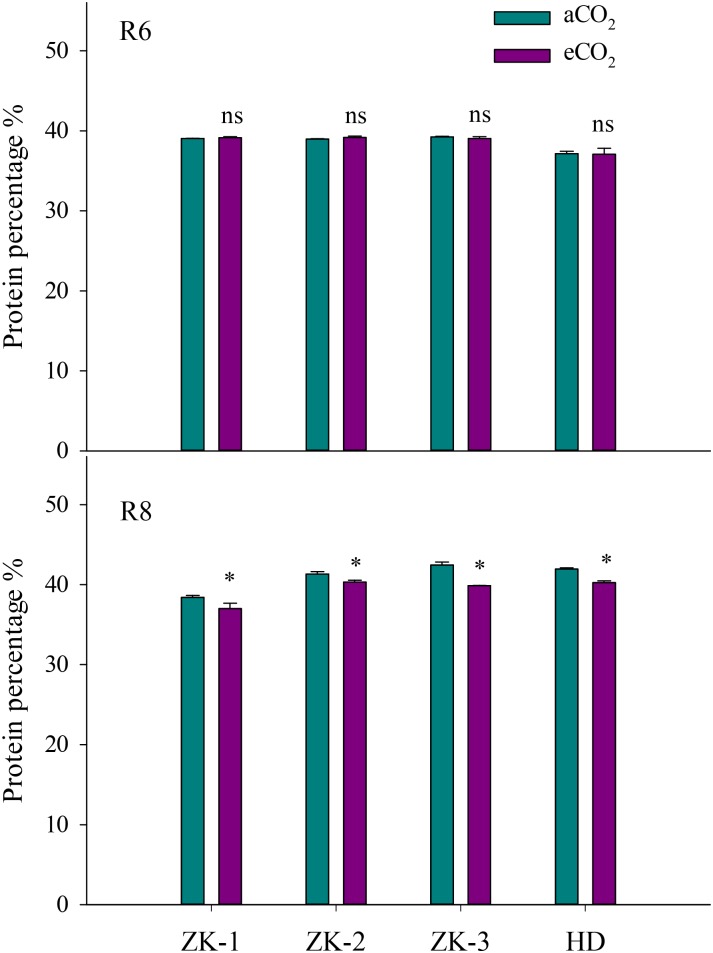
Influence of elevated CO_2_ on soybean seed protein concentrations at fresh edible (R6) and mature stages (R8) in the four soybean cultivars: Zhongke-maodou 1(ZK-1), Zhongke-maodou 2 (ZK-2), Zhongke-maodou 3(ZK-3), and Hei-maodou (HD); error bars represent the standard error (*n* = 3). Significant differences between CO_2_ treatments for each cultivar are noted with an asterisk at *P* < 0.05.

**FIGURE 2:**
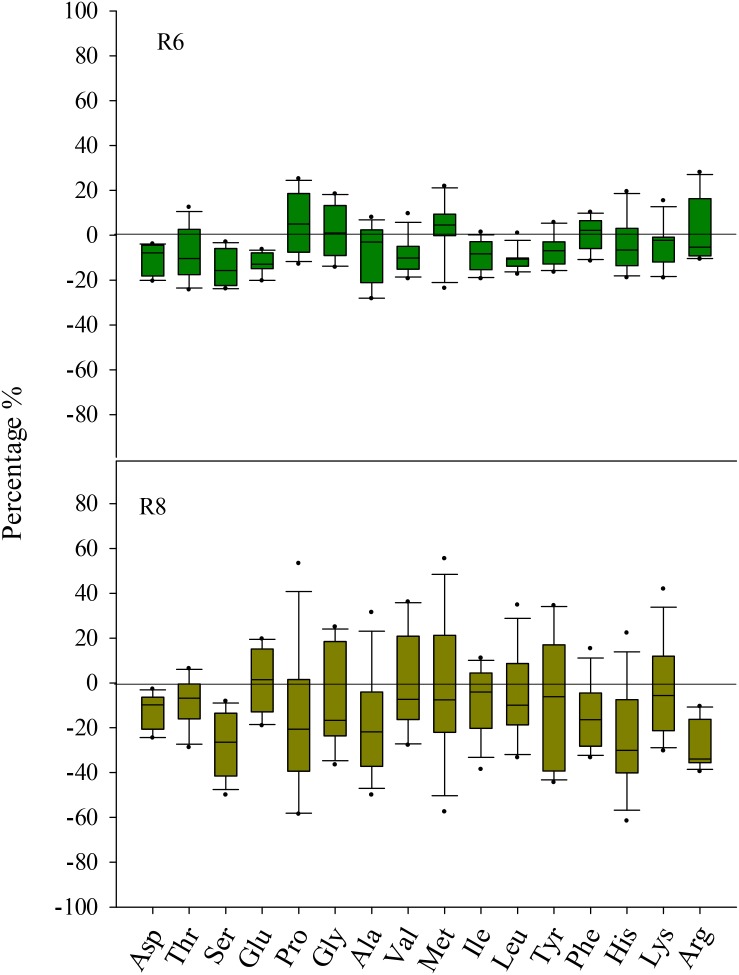
Boxplot shows the mean response ratio of the seed free amino acid concentrations of four soybean cultivars under elevated CO_2_ at fresh edible (R6) and mature stages (R8).

At R6, eCO_2_ had no influence on the protein concentration when compared with aCO_2_ (*P* > 0.05; **Figure 1**). However, eCO_2_ significantly (*P* < 0.05) decreased the free amino acid concentration at R6 (**Supplementary Table S1**). Except for Met, the concentrations of all other free amino acid decreased under eCO_2_ (*P* < 0.05). The extent of change in free amino acid concentrations in the seed in response to eCO_2_ varied between cultivars (*P* < 0.05; **Figure 2**), implying a significant CO_2_ × cultivar interaction (*P* < 0.05; **Table 1**).

**Table 1:** Significance levels of main effects and interactions of CO_2_ and cultivars on soybean grain free amino acid concentrations at R6 and R8.

	R6			R8		
	CO_2_	Genotype	C × G	CO_2_	Genotype	C × G
Asp	^∗∗∗^	^∗∗∗^	^∗∗∗^	^∗∗∗^	^∗∗∗^	^∗∗∗^
Thr	^∗∗∗^	^∗∗∗^	^∗∗∗^	n.s.	^∗∗∗^	n.s.
Ser	^∗∗∗^	^∗∗∗^	^∗∗∗^	^∗∗∗^	^∗∗∗^	^∗∗∗^
Glu	^∗∗∗^	^∗∗∗^	^∗∗∗^	n.s.	^∗∗∗^	^∗∗∗^
Pro	^∗∗^	^∗∗∗^	^∗∗∗^	^∗∗∗^	^∗∗∗^	^∗∗∗^
Gly	n.s.	^∗∗∗^	^∗∗^	n.s.	n.s.	n.s.
Ala	^∗∗∗^	^∗∗∗^	^∗∗∗^	^∗∗∗^	^∗∗∗^	n.s.
Val	^∗∗∗^	^∗∗∗^	^∗^	n.s.	^∗∗∗^	n.s.
Met	n.s.	^∗∗∗^	n.s.	n.s.	n.s.	n.s.
Ile	^∗∗∗^	^∗∗∗^	^∗∗∗^	n.s.	^∗∗^	n.s.
Leu	^∗∗∗^	^∗∗∗^	n.s.	n.s.	^∗∗∗^	n.s.
Tyr	^∗∗∗^	^∗∗∗^	n.s.	n.s.	n.s.	n.s.
Phe	n.s.	^∗∗∗^	^∗∗∗^	^∗∗^	n.s.	n.s.
His	n.s.	^∗∗∗^	^∗∗∗^	^∗∗∗^	^∗∗∗^	n.s.
Lys	^∗^	^∗∗∗^	^∗∗^	n.s.	^∗∗∗^	n.s.
Arg	n.s.	^∗∗∗^	^∗∗∗^	^∗∗∗^	^∗∗∗^	^∗∗∗^
Total	^∗∗∗^	^∗∗∗^	^∗∗∗^	^∗∗∗^	^∗∗∗^	^∗∗∗^

*^∗^P < 0.05; ^∗∗^P < 0.01; ^∗∗∗^P < 0.001; n.s., not significant (two-way ANOVA).*

At R8, eCO_2_ significantly decreased seed protein concentration by 3.6%, 2.4%, 4.1%, and 6.1% in ZK-1, ZK-2, ZK-3, and HD, respectively. A significant effect of CO_2_ × cultivar interaction on protein concentration was observed (*P* < 0.001; **Figure 1**). Correspondingly, the free amino acid concentration decreased significantly (*P* < 0.05) under eCO_2_. The concentrations of Glu and Pro increased in response to eCO_2_ in ZK-1, whereas the concentration of Lys increased in response to eCO_2_ in ZK-2 and ZK-3. There was no influence of eCO_2_ on the concentrations of Thr, Gly, Val, Met, Ile, Leu, and Tyr (*P* > 0.05) in any of the cultivars (**Table 1** and **Supplementary Table S2**).

### Oil and Fatty Acids

**FIGURE 3:**
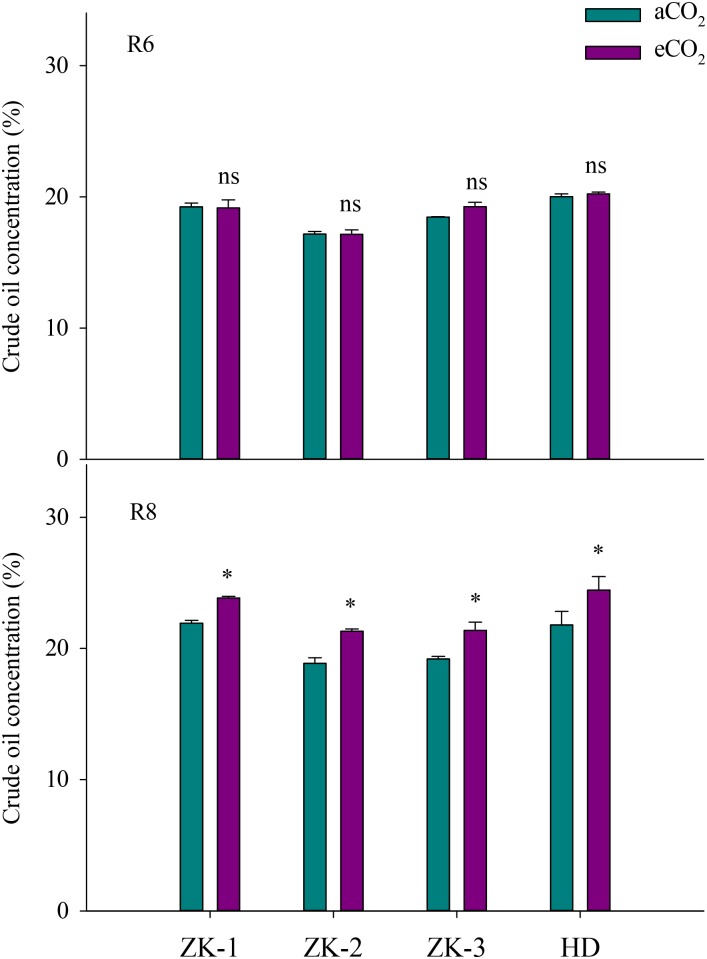
Influence of elevated CO_2_ on soybean seed oil concentrations at fresh edible (R6) and mature stages (R8) of four soybean cultivars: Zhongke-maodou 1 (ZK-1), Zhongke-maodou 2 (ZK-2), Zhongke-maodou 3 (ZK-3), and Hei-maodou (HD); error bars represent the standard error (*n* = 3). Significant differences between CO_2_ treatments for each cultivar are noted with an asterisk at *P* < 0.05.

**FIGURE 4:**
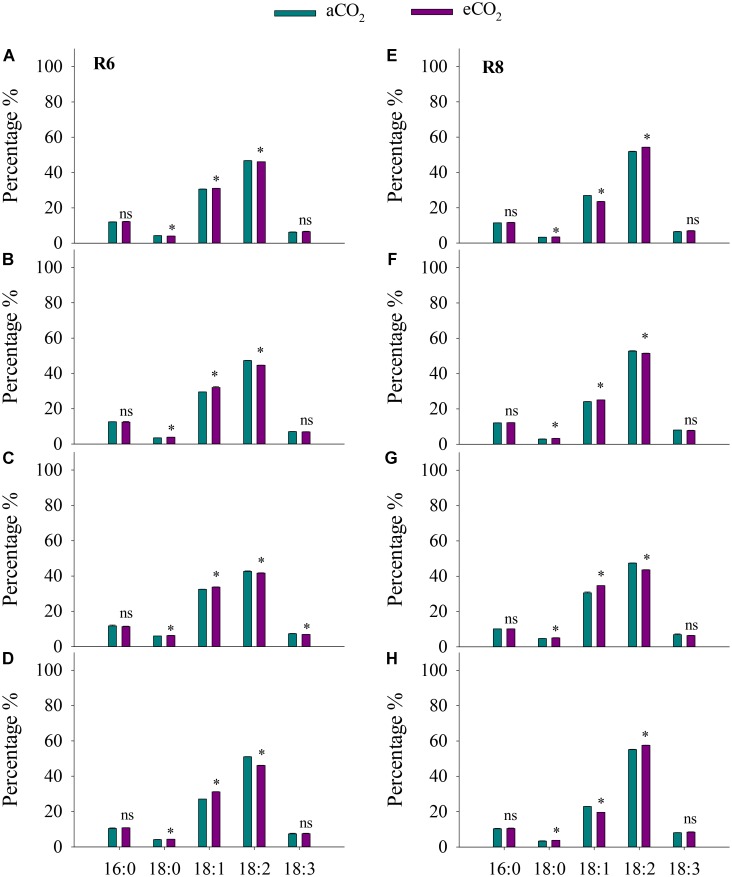
Influence of elevated CO_2_ on the percentage of palmitic (16:0), stearic acid (18:0), oleic acid (18:1), linoleic acid (18:2), and linoleic (18:3) acid of ZK-1 **(A)**, ZK-2 **(B)**, ZK-3 **(C)**, and HD **(D)** at the fresh edible stage (R6) and ZK-1 **(E)**, ZK-2 **(F)**, ZK-3 **(G)**, and HD **(H)** at the mature stage (R8); error bars represent the standard error (*n* = 3). Significant differences between CO_2_ treatments for each cultivar are noted with an asterisk at *P* < 0.05.

Elevated CO_2_ had no influence on soybean oil concentration in all cultivars at R6 (**Figure 3**). However, eCO_2_ significantly (*P* < 0.05) increased oleic acid (18:1) concentration by 1.4%, 8.5%, 3.8%, and 15% in ZK-1, ZK-2, ZK-3, and HD, respectively, and significantly decreased linoleic acid (18:2) concentration by 1.4%, 5.5%, 2.1%, and 9.6% (*P* < 0.05). Together, oleic acid and linoleic acid accounted for more than 75% of the total oil content. Furthermore, eCO_2_ significantly (*P* < 0.05) increased stearic acid (18:0) levels by 10%, 6.1%, and 4.8%, respectively, in ZK-2, ZK-3, and HD; however, this was significantly decreased by 6.3% in ZK-1 (*P* < 0.05). Elevated CO_2_ had no effect on either palmitic acid (16:0) or linoleic acid (18:3) concentration at R6 (*P* > 0.05; **Figure 4**).

At R8, eCO_2_ significantly increased (*P* < 0.05) seed oil concentration by 8.7%, 13%, 11%, and 12% in ZK-1, ZK-2, ZK-3, and HD, respectively (**Figure 3**). The content of oil significantly increased as well (**Supplementary Table S3**). Elevated CO_2_ increased stearic acid (18:0) concentration (*P* < 0.05) by 5.4%, 13%, 7.8%, and 12% in ZK-1, ZK-2, ZK-3, and HD, respectively. Differential responses of oleic acid (18:1) concentration to eCO_2_ among cultivars were found. Elevated CO_2_ significantly (*P* < 0.05) increased oleic acid (18:1) concentration by 4.2% and 13% in ZK-2 and ZK-3 but not in ZK-1 and HD. On the contrary, the concentration of linoleic acid (18:2) was (*P* < 0.05) decreased by 2.3% and 8.1% in ZK-2 and ZK-3, but it increased (*P* < 0.05) by 4.7% and 4.5% in ZK-1 and HD under eCO_2_ (**Figure 4**).

### Seed Isoflavones

**FIGURE 5:**
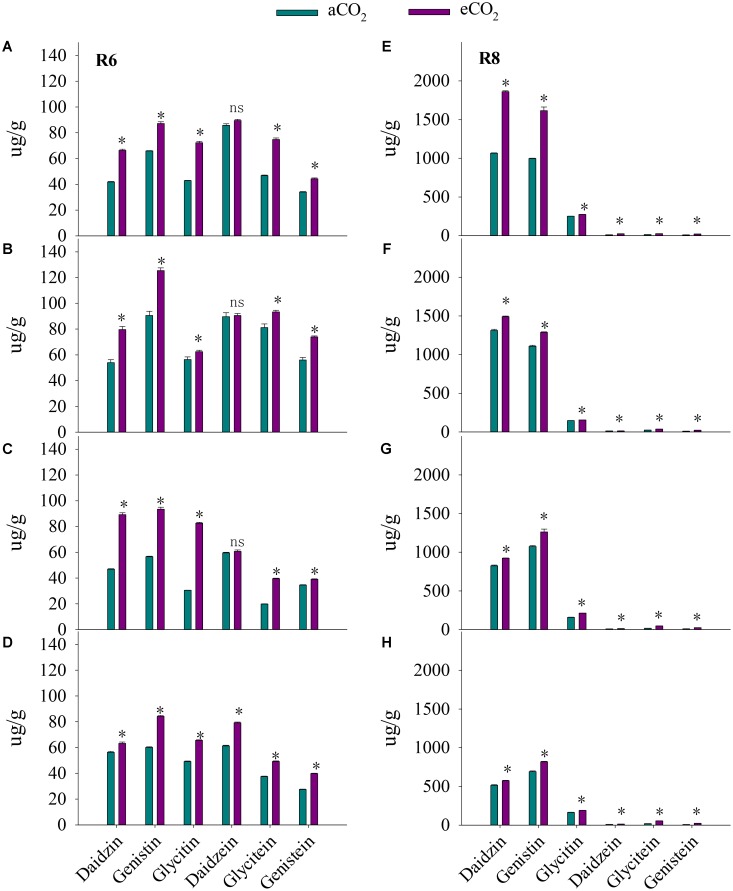
Influence of elevated CO_2_ on isoflavone concentrations of ZK-1 **(A)**, ZK-2 **(B)**, ZK-3 **(C)**, and HD **(D)** at the fresh edible stage (R6) and ZK-1 **(E)**, ZK-2 **(F)**, ZK-3 **(G)**, and HD **(H)** at the mature stage (R8); error bars represent the standard error (*n* = 3). Significant differences between CO_2_ treatments for each cultivar are noted with an asterisk at *P* < 0.05.

Compared with aCO_2_, at R6, eCO_2_ increased total isoflavone concentration by 38% across the four cultivars (**Figure 5**). The increase in isoflavone concentration in response to eCO_2_ varied among cultivars (*P* < 0.001), implying a significant CO_2_ × cultivar interaction (*P* < 0.001). The concentrations of daidzin, glycitin, genistin, glycitein, and genistein were significantly (*P* < 0.01) increased in all cultivars in response to eCO_2_. Elevated CO_2_ increased the concentration of daidzein by 29% in HD (*P* < 0.05) but not in ZK-1, ZK-2, and ZK-3.

At R8, eCO_2_ significantly increased total isoflavone concentration by 28% across the four cultivars. Significant increases in the concentrations of daidzein, daidzin, glycitin, genistin, glycitein, and genistein were observed (*P* < 0.001) in all cultivars under eCO_2_ (**Figure 5**).

### Mineral Element Concentrations

**FIGURE 6:**
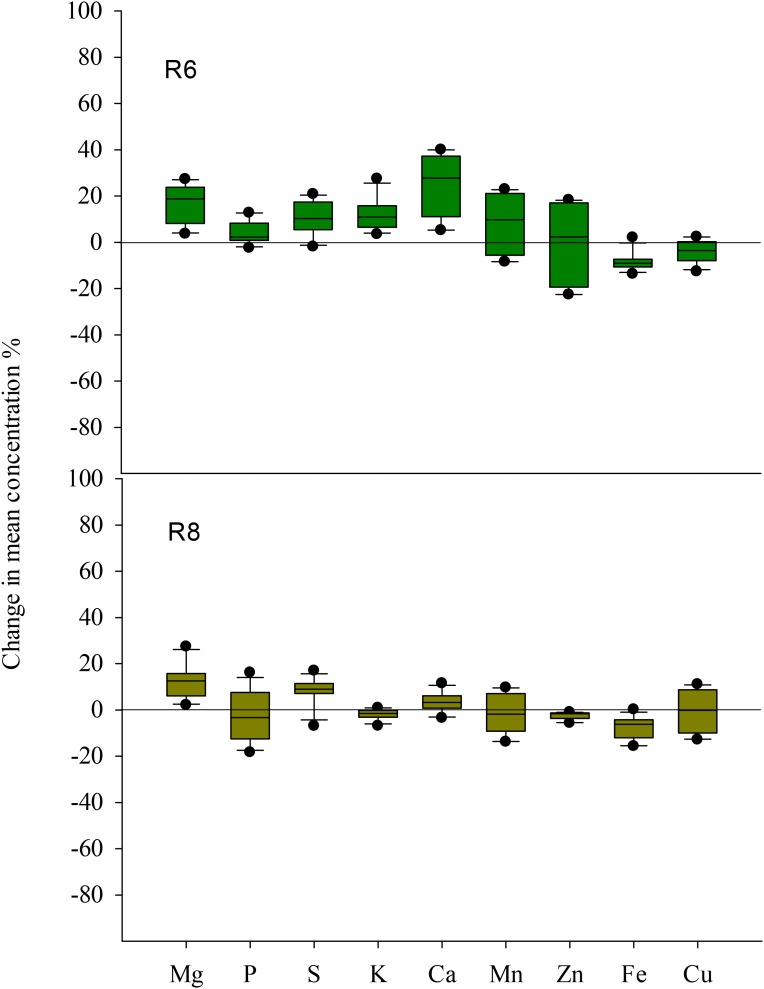
Boxplot shows the mean response ratio of the grain chemical element concentrations of four soybean cultivars under elevated CO_2_ at the fresh edible (R6) and mature stages (R8).

Elevated CO_2_ had no influence on Cu concentration in soybean seed at R6 (**Table 2**). However, the concentrations of other mineral elements responded differently to eCO_2_ among cultivars. Under eCO_2_, the Zn concentration was significantly decreased by 10% and 22% in ZK-1 and ZK-3, respectively, but significantly increased (*P* < 0.05) by 14% and 18% in ZK-2 and HD, respectively (**Table 2** and **Figure 6**). The Fe concentration was decreased by 10%, 8.2%, 5.9%, and 13% (*P* < 0.05) in ZK-1, ZK-2, ZK-3, and HD, respectively (**Table 2** and **Figure 6**). The Mn concentration was also decreased significantly by 8.3% in ZK-3 under eCO_2_ but increased in the other three cultivars. Elevated CO_2_ increased the P concentration by 12% in HD, whereas no changes were observed in other cultivars. The S concentration was increased by 10%, 13%, and 19% (*P* < 0.05) in ZK-1, ZK-2, and HD, respectively, under eCO_2_, whereas the concentration remained unchanged in ZK-3. Elevated CO_2_ increased Ca concentration (*P* < 0.05) by 23% and 34% in ZK-3 and HD, respectively, but it had no influence on Ca concentration in ZK-1 and ZK-2 (**Table 3**). Although Mg concentration changed only slightly in HD, a significant (*P* < 0.05) increase in ZK-1, ZK-2, and ZK-3 was found under eCO_2_. Consistent increases in K concentration among cultivars were found under eCO_2_, with 13%, 22%, 7.4%, and 6.1% increase in ZK-1, ZK-2, ZK-3, and HD, respectively (*P* < 0.05).

**Table 2:** Effect of eCO_2_ on soybean seed element concentrations at R6 and R8.

		R6		R8	
Cultivar		aCO_2_	eCO_2_	aCO_2_	eCO_2_
Mg	ZK-1	4.96	5.93^∗∗^	6.39	7.31^∗∗^
mg/g	ZK-2	5.66	6.96^∗^	5.65	6.09^n.s.^
	ZK-3	4.96	6.03^∗^	5.96	6.53^n.s.^
	HD	6.96	7.30^∗^	7.41	8.75^∗^
P	ZK-1	9.60	9.70^n.s.^	14.65	12.72^n.s.^
mg/g	ZK-2	8.77	8.96^n.s.^	11.40	12.17^n.s.^
	ZK-3	7.79	7.87^n.s.^	10.34	10.29^n.s.^
	HD	8.45	9.44^∗∗^	13.15	12.48^n.s.^
S	ZK-1	6.61	7.28^∗^	7.87	7.92^n.s.^
mg/g	ZK-2	5.25	5.94^∗^	8.02	8.98^∗∗^
	ZK-3	6.27	6.32^n.s.^	7.79	8.63^∗^
	HD	6.21	7.42^∗∗^	8.27	9.00^∗^
K	ZK-1	25.0	28.2^∗∗^	34.3	34.1^n.s.^
mg/g	ZK-2	20.7	25.2^∗^	30.8	30.6^n.s.^
	ZK-3	24.5	26.3^n.s.^	32.3	32.1^n.s.^
	HD	24.9	26.4^∗∗^	35.9	34.1^n.s.^
Ca	ZK-1	3.77	4.64^n.s.^	5.50	5.68^n.s.^
mg/g	ZK-2	3.29	3.60^n.s.^	5.10	5.42^∗∗^
	ZK-3	4.07	5.45^∗∗^	3.82	3.85^n.s.^
	HD	3.27	4.36^∗∗^	4.52	4.63^n.s.^
Mn	ZK-1	20.4	23.9^∗∗^	16.9	18.4^∗∗^
ug/g	ZK-2	15.5	15.9^∗∗^	14.6	14.8^∗∗^
	ZK-3	19.9	18.2^∗∗^	18.4	16.1^∗∗^
	HD	20.2	24.8^∗∗^	29.1	27.6^∗∗^
Zn	ZK-1	36.6	33.1^∗∗^	43.3	42.7^∗^
ug/g	ZK-2	35.3	40.2^∗∗^	43.2	42.7^n.s.^
	ZK-3	38.0	29.4^∗∗^	42.2	41.4^∗∗^
	HD	35.3	41.7^∗∗^	44.8	42.6^∗∗^
Fe	ZK-1	49.8	45.2^∗∗^	63.4	59.5^∗^
ug/g	ZK-2	51.5	47.6^∗∗^	63.8	59.7^∗^
	ZK-3	45.2	42.7^∗^	55.1	53.2^∗^
	HD	45.1	39.9^∗∗^	54.1	46.2^∗^
Cu	ZK-1	22.9	20.5^n.s.^	23.1	25.5^∗∗^
ug/g	ZK-2	20.6	20.5^n.s.^	18.6	19.2^∗∗^
	ZK-3	23.2	21.6^n.s.^	24.4	23.5^∗^
	HD	20.2	20.5^n.s.^	25.9	22.7^∗^

*^∗^, ^∗∗^, and n.s. indicate significance at 0.05, 0.01 level, and non-significant difference (t-test) between aCO_2_ and eCO_2_, respectively, for individual cultivars.*

**Table 3:** Significance levels of main effects and interactions of CO_2_ and cultivars on soybean grain element nutrients concentrations at R6 and R8.

	R6			R8		
	CO_2_	Genotype	C × G	CO_2_	Genotype	C × G
Mg	^∗∗∗^	^∗∗∗^	n.s.	^∗∗∗^	^∗∗∗^	n.s.
P	^∗∗^	^∗∗∗^	^∗^	n.s.	^∗∗^	n.s.
S	^∗∗∗^	^∗∗∗^	^∗∗^	^∗∗^	^∗^	n.s.
K	^∗∗∗^	^∗∗∗^	^∗∗^	^∗^	^∗∗∗^	^∗∗^
Ca	^∗∗∗^	^∗∗∗^	n.s.	^∗^	^∗∗∗^	n.s.
Mn	^∗∗∗^	^∗∗∗^	^∗∗∗^	^∗∗^	^∗∗∗^	^∗∗∗^
Zn	^∗^	^∗∗∗^	^∗∗∗^	^∗∗^	^∗∗∗^	^∗∗∗^
Fe	^∗^	^∗∗∗^	^∗∗∗^	^∗∗∗^	^∗∗∗^	n.s.
Cu	n.s.	^∗∗^	n.s.	n.s.	^∗∗∗^	^∗∗∗^

*^∗^P < 0.05; ^∗∗^P < 0.01; ^∗∗∗^P < 0.001; n.s., not significant (two-way ANOVA).*

At R8, eCO_2_ had no effect on P and K concentrations in seeds (**Tables 2**, **3**). Elevated CO_2_ had a significantly positive effect on Mg, S, and Ca concentrations (**Figure 6**). On the contrary, Zn and Fe concentrations were significantly (*P* < 0.05) decreased under eCO_2_. The Mn concentration was decreased by 12% and 5.2% in ZK-3 and HD, respectively, but significantly increased by 9.3% and 1.7% in ZK-1 and ZK-2, respectively, under eCO_2_. Similarly, the Cu concentration was decreased by 3.8% and 14% in ZK-3 and HD, respectively, but significantly increased by 10% and 3.2% in ZK-1 and ZK-2, respectively, under eCO_2_ (**Table 2**). The protein and nutrient contents of the four cultivars increased under eCO_2_ compared with aCO_2_ (**Supplementary Table S3**).

## Discussion

This study demonstrated that eCO_2_ had no influence on protein concentration in soybean seed at R6, but eCO_2_ significantly (*P* < 0.05) decreased protein concentration at R8 (**Figure 1**). This finding suggests that, although soybean plants are able to symbiotically fix N_2_ to mitigate N deficiency, shortfalls still occur after R6 under eCO_2_ when grown in Mollisols. Several studies argue that the lower seed protein concentration under eCO_2_ is attributed to the dilution effect, as eCO_2_ increases the accumulation of carbohydrates (Gifford et al., 2000; Wu et al., 2004). The increased carbon (C) gain under eCO_2_ might be used for protein synthesis, which requires a large amount of energy for the maintenance of the synthetic process (Fangmeier et al., 2002; Pérez-López et al., 2014). In this context, the CO_2_-induced reduction in protein concentration cannot be fully attributed to N limitation in soybeans under eCO_2_. Several other authors also reported that the decrease in protein concentration under eCO_2_ could not be diminished by additional N supply (Fangmeier et al., 1997; Weigel and Manderscheid, 2005). Therefore, soybean crops grown under eCO_2_ may have lower protein content, with nutritional implications for humans and animals that consume these crops as a food source. Specialized breeding strategies that intend to enhance seed quality are needed to address this issue (Bellaloui et al., 2015).

In the present study, eCO_2_ lowered total free amino acid concentrations both at the R6 and R8 stages. This indicated that the nutrient values of total free amino acids at both stages were reduced under eCO_2_ (**Supplementary Table S1**). Free amino acids are an important form of N storage in soybean seed during the processes of N assimilation and protein synthesis (Takahashi et al., 2003; Song et al., 2013). In particular, glutamine is the only product into which inorganic N is transformed (Pratelli and Pilot, 2014). Arginine is important in protein synthesis, and it is predominantly derived from glutamine in the urea cycle (Takahashi et al., 2003)_._ Therefore, the decreased levels of free amino acids under eCO_2_ may be channeled toward the synthesis of functional proteins rather than those stored in the soybean seed.

The present study demonstrated that eCO_2_ significantly increased the oil concentration of soybeans at R8 (**Figure 3**); this result is supported by previous findings of an increase in oil concentration in soybean or other crops under eCO_2_ (Heagle et al., 1998; Högy et al., 2010; Hao et al., 2014). This phenomenon is reasonable, as oil synthesis and storage in plants, which are enhanced under eCO_2_, are involved in carbon and energy supply (Rawsthorne, 2002; Bates et al., 2013). Singh et al. (2016) also reported that the increased seed oil concentration under eCO_2_ is attributed to the direct stimulatory effect on photosynthesis. However, no studies have investigated oil composition in soybean under eCO_2_. To our knowledge, the present study is the first to report that eCO_2_ consistently increased oleic acid (18:1) concentrations but decreased linoleic acid (18:2) concentrations at R6 and R8. This finding indicates that eCO_2_ improves soybean oil quality, with potential benefits for human health. High levels of oleic acid (18:1) enhance the oxidative stability of soybean oil, giving it a longer shelf life (Clemente and Cahoon, 2009), whereas the increased levels of trans-fatty acids by partial hydrogenation of linoleic acid (18:2) are associated with heart disease (Demorest et al., 2016). Nevertheless, the mechanism underlying the increase in fatty acids under eCO_2_ is complex; major gaps remain in our understanding of the regulation of fatty acid synthesis, especially in tissues that store large amounts of oil (Rawsthorne, 2002; Bates et al., 2013).

The present study found that eCO_2_ significantly increased total and specific isoflavone concentrations at R6 and R8 (**Figure 5**). These results were in agreement with a previous study, which demonstrated that variation of isoflavones in soybeans was positively correlated with CO_2_ level (Kim et al., 2005). Theoretically, the synthesis of isoflavones via the phenylpropanoid pathway requires high levels of carbon (Ralston et al., 2005; Tsai et al., 2006). Weisshaar and Jenkins (1998) reported that approximately 20% of the carbon from photosynthesis is used to synthesize the phenolic compounds found in nature, including flavonoids and isoflavonoids. Larger amounts of carbon could be obtained to generate isoflavones from plants that were grown under eCO_2_ (Ainsworth et al., 2002; Kretzschmar et al., 2009; O’Neill et al., 2010). Therefore, environmental conditions, including CO_2_ level, strongly influence isoflavone concentration. Dhaubhadel et al. (2003) stated that the expression of two hydroxy isoflavanone synthase genes, *IFS1* and *IFS2*, in different tissues, is influenced by environmental conditions. Owing to the health-promoting effects of isoflavones on human vasomotor symptoms, the cardiovascular system, the breast, uterus, bone, and cognition, foods with high levels of isoflavone have been recommended by the U.S. Food and Drug Administration (FDA) (Morrison et al., 2008; Clarkson et al., 2011). In the present study, the increase in isoflavone concentration of soybean observed in response to eCO_2_ suggests improved nutritional value of soybean under the scenario of rising CO_2_ levels.

The biogeochemical cycles of nutrients are affected by eCO_2_, and the resulting changes in seed nutrient concentration pose a potential challenge to human health. In this study, eCO_2_ greatly lowered the nutritional value of seed in terms of Zn and Fe content (**Table 2**); similar results have been found in rice, wheat, and barley (Myers et al., 2014; Zhu et al., 2018). This may increase the risk of micronutrient malnutrition and other related diseases. Several studies attribute this phenomenon to the dilution effect, which is caused by the increased growth of plants under eCO_2_. The eCO_2_-induced increase in grain nutrient content (**Supplementary Table S3**) also indicates that the demand for nutrients increases under such an environment, which may, to some extent, lead to dilution effect. As a result of the decrease in stomatal conductance under eCO_2_, plants tend to undergo reduced transpiration, leading to decreased mass flow (Rogers et al., 1999; Bunce, 2001) and absorption of mobile elements such as N (McDonald et al., 2002). However, P and K concentrations were not influenced by eCO_2_ in the present study (**Table 2**). This is likely because the increase in soil moisture due to reduced transpiration under eCO_2_ is beneficial for the diffusion of specific elements in soil to the roots, thus, increasing their availability. Nevertheless, the two primary mechanisms fail to explain the responses of all elements in seeds to eCO_2_, as significant increases in Mg, S, and Ca concentrations, or no change in Cu concentration, were found in this study (**Figure 6**). Pérez-López et al. (2014) reported that the increase in growth under eCO_2_ could be attributed to the stimulation of metabolic activity in plants, and, accordingly, to the requirement of nutrients that serve as enzyme cofactors in metabolic reactions (Ca, Mg, and Mn) and redox reactions (Fe, Zn, and Cu). Therefore, eCO_2_ has both positive and negative effects on the nutritional quality of soybean seeds. However, further study of the mechanism by which eCO_2_ influences seed nutrients is necessary, not only because the different elements show varying responses to eCO_2_ at the same growth stage but also because the same element shows differential responses at R6 and R8.

**FIGURE 7:**
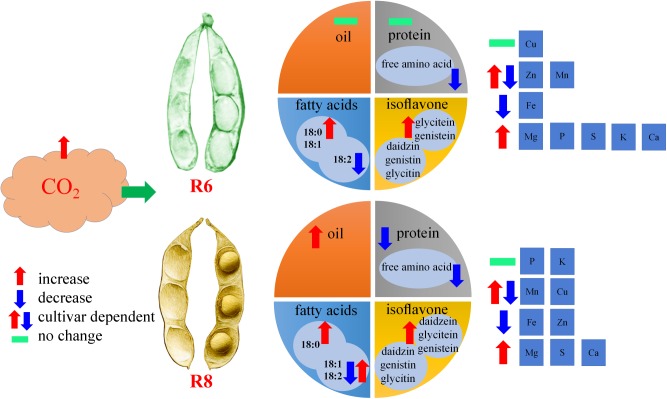
Diagram illustrating the impact of elevated CO_2_ on seed quality of soybean at the fresh edible (R6) and mature stages (R8).

In summary, protein concentration in soybean seeds was significantly decreased under eCO_2_; however, oil concentration showed the opposite trend at R8. The free amino acid concentration was significantly decreased under eCO_2_, irrespective of the growth stage. Elevated CO_2_ resulted in an increase in oleic acid concentration (18:1) of all cultivars at R6. Total isoflavone concentrations were significantly increased at R6 and R8. The concentrations of Fe were significantly decreased at R6 and R8 under eCO_2_, while the changes in Zn and Mn concentrations varied among cultivars (**Figure 7**). These results suggest that eCO_2_ may promote fat content by enhancing oleic acid levels (18:1) but decrease the content of proteins and relevant amino acids.

## Author Contributions

JJ and YL designed the experiments and managed the projects. YL, ZY, CL, QZ, JW, and CW performed the experiments. YL, JJ, GW, and XL performed the data analysis. YL, JJ, and XL wrote the manuscript.

## Conflict of Interest Statement

The authors declare that the research was conducted in the absence of any commercial or financial relationships that could be construed as a potential conflict of interest. The reviewer PL and handling editor declared their shared affiliation at time of review.
