# Supplementary material for: Impact of Elevated CO2 on Seed Quality of Soybean at the Fresh Edible and Mature Stages
Source: Front Plant Sci. 2018 Oct 17;9:1413. doi: 10.3389/fpls.2018.01413 (PMC6199416; doi:10.3389/fpls.2018.01413)
Supplement: Supplementary file 1 [file Table_1.DOCX]

**TABLE S1 ∣** Effect of eCO_2_ on soybean seed free amino acids concentrations at R6 (mean ± SE, mg g^-1^).

|  | ZK-1 | | ZK-2 | | ZK-3 | | HD | |
| --- | --- | --- | --- | --- | --- | --- | --- | --- |
|  | aCO_2_ | eCO_2_ | aCO_2_ | eCO_2_ | CO_2_ | CO_2_ | CO_2_ | eCO_2_ |
| Asp | 2.31±0.012 | 1.86±0.014 ^**^ | 2.40±0.008 | 2.10±0.035 ^**^ | 4.26±0.006 | 4.09±0.005 ^**^ | 2.25±0.008 | 2.12±0.004 ^**^ |
| Thr | 0.61±0.012 | 0.53±0.007 ^**^ | 0.65±0.020 | 0.69±0.005 ^n.s.^ | 0.65±0.012 | 0.62±0.008 ^n.s.^ | 0.73±0.007 | 0.57±0.006 ^**^ |
| Ser | 1.37±0.020 | 1.07±0.003 ^**^ | 1.44±0.009 | 1.38±0.001 ^**^ | 2.61±0.008 | 2.35±0.018 ^**^ | 1.79±0.013 | 1.39±0.004 ^**^ |
| Glu | 0.53±0.001 | 0.48±0.009 ^**^ | 2.31±0.003 | 1.99±0.004 ^**^ | 0.41±0.003 | 0.34±0.005 ^**^ | 0.90±0.013 | 0.83±0.007 ^**^ |
| Pro | 0.44±0.006 | 0.54±0.006 ^**^ | 0.74±0.004 | 0.66±0.009 ^**^ | 0.95±0.008 | 0.90±0.007 ^*^ | 0.63±0.007 | 0.72±0.003 ^**^ |
| Gly | 0.29±0.004 | 0.26±0.010 ^n.s.^ | 0.36±0.013 | 0.37±0.014 ^n.s.^ | 0.36±0.003 | 0.33±0.014 ^n.s.^ | 0.25±0.007 | 0.30±0.010 ^*^ |
| Ala | 0.74±0.007 | 0.54±0.007 ^**^ | 1.21±0.013 | 1.27±0.006 ^*^ | 1.53±0.013 | 1.50±0.004 ^n.s.^ | 0.72±0.006 | 0.68±0.010 ^*^ |
| Val | 0.88±0.006 | 0.73±0.009 ^**.^ | 0.91±0.010 | 0.90±0.058 ^n.s.^ | 1.09±0.005 | 0.98±0.007 ^**^ | 0.89±0.006 | 0.83±0.011 ^*^ |
| Met | 0.02±0.010 | 0.03±0.005 ^n.s.^ | 0.02±0.007 | 0.03±0.000 ^n.s.^ | 0.07±0.007 | 0.08±0.006 ^n.s.^ | 0.03±0.009 | 0.03±0.013 ^n.s.^ |
| Ile | 0.65±0.009 | 0.53±0.007 ^**.^ | 0.69±0.005 | 0.68±0.011 ^n.s.^ | 0.81±0.014 | 0.73±0.011 ^*^ | 0.67±0.013 | 0.63±0.010 ^n.s.^ |
| Leu | 0.96±0.009 | 0.82±0.006 ^**.^ | 1.05±0.012 | 0.96±0.061 ^n.s.^ | 1.13±0.010 | 1.01±0.010 ^**^ | 1.23±0.006 | 1.11±0.008 ^**^ |
| Tyr | 0.77±0.011 | 0.68±0.013 ^**.^ | 0.59±0.024 | 0.58±0.017 ^n.s.^ | 0.55±0.012 | 0.50±0.006 ^*^ | 0.75±0.008 | 0.71±0.003 ^*^ |
| Phe | 1.64±0.011 | 1.49±0.010 ^**.^ | 1.43±0.005 | 1.56±0.005 ^n.s.^ | 1.94±0.008 | 1.99±0.005 ^**^ | 1.09±0.004 | 1.09±0.008 ^n.s.^ |
| His | 0.47±0.003 | 0.42±0.006 ^**.^ | 0.49±0.028 | 0.57±0.010 ^*^ | 0.64±0.013 | 0.61±0.012 ^n.s.^ | 0.44±0.004 | 0.37±0.010 ^**^ |
| Lys | 0.47±0.018 | 0.42±0.009 ^n.s.^ | 0.53±0.007 | 0.57±0.018 ^n.s.^ | 0.49±0.003 | 0.43±0.016 ^*^ | 0.68±0.008 | 0.66±0.010 ^n.s.^ |
| Arg | 0.83±0.012 | 0.77±0.019 ^n.s.^ | 1.38±0.015 | 1.27±0.012 ^*^ | 1.12±0.009 | 1.05±0.008 ^**^ | 1.17±0.010 | 1.47±0.009 ^**^ |
| Total | 13.0±0.01 | 11.2±0.06 ^**.^ | 16.2±0.09 | 15.6±0.17 ^*^ | 18.6±0.08 | 17.5±0.02 ^**^ | 14.2±0.04 | 13.5±0.01 ^**^ |

*, ** and n.s. indicate significant at 0.05, 0.01 level and non-significant difference (t-test) between aCO_2_ and eCO_2_, respectively, for individual cultivars.

**TABLE S2 ∣** Effect of eCO_2_ on soybean seed free amino acids concentrations at R8 (mean ± SE, mg g^-1^).

|  | ZK-1 | | ZK-2 | | ZK-3 | | HD | |
| --- | --- | --- | --- | --- | --- | --- | --- | --- |
|  | aCO_2_ | eCO_2_ | aCO_2_ | eCO_2_ | CO_2_ | CO_2_ | CO_2_ | eCO_2_ |
| Asp | 0.58±0.003 | 0.55±0.010 ^*^ | 0.86±0.012 | 0.76±0.007 ^**^ | 1.48±0.011 | 1.13±0.003 ^**^ | 0.68±0.009 | 0.63±0.009 ^*^ |
| Thr | 0.14±0.005 | 0.13±0.014 ^n.s.^ | 0.16±0.009 | 0.15±0.005 ^n.s.^ | 0.22±0.006 | 0.22±0.007 ^n.s.^ | 0.15±0.014 | 0.12±0.011 ^n.s.^ |
| Ser | 0.18±0.007 | 0.14±0.005 ^**^ | 0.26±0.004 | 0.23±0.002 ^**^ | 0.56±0.003 | 0.32±0.001 ^**^ | 0.26±0.013 | 0.15±0.007 ^**^ |
| Glu | 0.60±0.006 | 0.71±0.009 ^**^ | 0.97±0.016 | 1.01±0.010 ^n.s.^ | 0.88±0.003 | 0.73±0.006 ^**^ | 0.53±0.007 | 0.52±0.014 ^n.s.^ |
| Pro | 0.04±0.008 | 0.05±0.007 ^n.s.^ | 0.06±0.004 | 0.05±0.001 ^n.s.^ | 0.23±0.019 | 0.10±0.006 ^**^ | 0.06±0.006 | 0.04±0.013 ^n.s.^ |
| Gly | 0.06±0.010 | 0.07±0.012 ^n.s.^ | 0.07±0.003 | 0.07±0.010 ^n.s.^ | 0.09±0.034 | 0.08±0.004 ^n.s.^ | 0.06±0.007 | 0.05±0.006 ^n.s.^ |
| Ala | 0.13±0.003 | 0.13±0.009 ^n.s.^ | 0.16±0.005 | 0.10±0.009 ^**^ | 0.17±0.019 | 0.13±0.001 ^n.s.^ | 0.09±0.008 | 0.07±0.012 ^n.s.^ |
| Val | 0.10±0.003 | 0.07±0.012 ^n.s.^ | 0.08±0.007 | 0.09±0.007 ^n.s.^ | 0.14±0.003 | 0.15±0.013 ^n.s.^ | 0.11±0.008 | 0.09±0.004 ^n.s.^ |
| Met | 0.02±0.006 | 0.01±0.007 ^n.s.^ | 0.01±0.001 | 0.01±0.002 ^n.s.^ | 0.03±0.008 | 0.03±0.004 ^n.s.^ | 0.02±0.002 | 0.01±0.003 ^n.s.^ |
| Ile | 0.05±0.003 | 0.05±0.006 ^n.s.^ | 0.04±0.004 | 0.04±0.003 ^n.s.^ | 0.05±0.001 | 0.05±0.003 ^n.s.^ | 0.07±0.010 | 0.05±0.008 ^n.s.^ |
| Leu | 0.08±0.005 | 0.07±0.009 ^n.s.^ | 0.05±0.004 | 0.06±0.012 ^n.s.^ | 0.10±0.006 | 0.10±0.008 ^n.s.^ | 0.08±0.010 | 0.06±0.013 ^n.s.^ |
| Tyr | 0.06±0.006 | 0.05±0.005 ^n.s.^ | 0.07±0.018 | 0.07±0.007 ^n.s.^ | 0.08±0.007 | 0.08±0.018 ^n.s.^ | 0.07±0.004 | 0.06±0.010 ^n.s.^ |
| Phe | 0.05±0.011 | 0.04±0.014 ^n.s.^ | 0.06±0.008 | 0.05±0.004 ^n.s.^ | 0.09±0.007 | 0.05±0.008 ^*^ | 0.06±0.009 | 0.05±0.004 ^n.s.^ |
| His | 0.12±0.007 | 0.08±0.009 ^*^ | 0.24±0.008 | 0.18±0.019 ^*^ | 0.11±0.000 | 0.10±0.001 ^**^ | 0.11±0.014 | 0.07±0.014 ^n.s.^ |
| Lys | 0.10±0.006 | 0.09±0.007 ^n.s.^ | 0.12±0.008 | 0.13±0.014 ^n.s.^ | 0.16±0.011 | 0.18±0.012 ^n.s.^ | 0.13±0.009 | 0.10±0.004 ^*^ |
| Arg | 0.94±0.020 | 0.62±0.032 ^**^ | 3.32±0.005 | 2.95±0.019 ^**^ | 1.71±0.004 | 1.13±0.008 ^**^ | 0.86±0.013 | 0.55±0.005 ^**^ |
| Total | 3.25±0.06 | 2.85±0.08 ^*^ | 6.25±0.02 | 5.97±0.03 ^**^ | 6.09±0.04 | 4.59±0.08 ^**^ | 3.31±0.05 | 2.65±0.04 ^**^ |

*, ** and n.s. indicate significant at 0.05, 0.01 level and non-significant difference (t-test) between aCO_2_ and eCO_2_, respectively, for individual cultivars.

**TABLE S3 ∣** Effect of eCO_2_ on soybean seed element, oil and protein contents, at R6 and R8.

|  |  | R6 | | | R8 | | |
| --- | --- | --- | --- | --- | --- | --- | --- |
| Cultivar | | aCO_2_ | eCO_2_ | % | aCO_2_ | eCO_2_ | % |
| Mg | ZK-1 | 70 | 114 | 63 | 117 | 178 | 53 |
| mg/plant | ZK-2 | 79 | 137 | 72 | 97 | 141 | 46 |
|  | ZK-3 | 65 | 111 | 72 | 111 | 153 | 37 |
|  | HD | 76 | 97 | 27 | 119 | 182 | 53 |
| P | ZK-1 | 135 | 187 | 38 | 267 | 310 | 16 |
| mg/plant | ZK-2 | 123 | 176 | 43 | 195 | 282 | 45 |
|  | ZK-3 | 102 | 144 | 42 | 192 | 240 | 25 |
|  | HD | 92 | 125 | 35 | 212 | 263 | 24 |
| S | ZK-1 | 93 | 140 | 50 | 144 | 193 | 34 |
| mg/plant | ZK-2 | 82 | 117 | 41 | 137 | 209 | 52 |
|  | ZK-3 | 82 | 116 | 42 | 145 | 200 | 38 |
|  | HD | 68 | 98 | 44 | 133 | 189 | 42 |
| K | ZK-1 | 353 | 544 | 54 | 624 | 841 | 35 |
| mg/plant | ZK-2 | 290 | 469 | 61 | 529 | 710 | 34 |
|  | ZK-3 | 319 | 485 | 52 | 603 | 750 | 25 |
|  | HD | 272 | 349 | 28 | 579 | 721 | 24 |
| Ca | ZK-1 | 53 | 89 | 68 | 100 | 138 | 38 |
| mg/plant | ZK-2 | 46 | 71 | 54 | 87 | 118 | 35 |
|  | ZK-3 | 53 | 100 | 89 | 71 | 88 | 24 |
|  | HD | 36 | 58 | 61 | 72 | 97 | 35 |
| Mn | ZK-1 | 0.29 | 0.46 | 60 | 0.31 | 0.45 | 46 |
| ug/plant | ZK-2 | 0.22 | 0.31 | 44 | 0.25 | 0.34 | 38 |
|  | ZK-3 | 0.26 | 0.34 | 30 | 0.34 | 0.38 | 10 |
|  | HD | 0.22 | 0.33 | 48 | 0.47 | 0.58 | 24 |
| Zn | ZK-1 | 0.52 | 0.64 | 23 | 0.75 | 1.07 | 41 |
| ug/plant | ZK-2 | 0.49 | 0.79 | 60 | 0.74 | 0.99 | 34 |
|  | ZK-3 | 0.50 | 0.54 | 10 | 0.79 | 0.97 | 23 |
|  | HD | 0.39 | 0.55 | 43 | 0.72 | 0.90 | 24 |
| Fe | ZK-1 | 0.70 | 0.87 | 24 | 1.16 | 1.45 | 25 |
| ug/plant | ZK-2 | 0.72 | 0.94 | 30 | 1.09 | 1.39 | 27 |
|  | ZK-3 | 0.59 | 0.97 | 65 | 1.03 | 1.24 | 20 |
|  | HD | 0.49 | 0.53 | 7 | 0.87 | 0.98 | 12 |
| Cu | ZK-1 | 0.32 | 0.40 | 23 | 0.42 | 0.62 | 47 |
| ug/plant | ZK-2 | 0.29 | 0.40 | 39 | 0.32 | 0.45 | 40 |
|  | ZK-3 | 0.30 | 0.40 | 31 | 0.45 | 0.55 | 21 |
|  | HD | 0.22 | 0.27 | 23 | 0.42 | 0.48 | 15 |
| Oil | ZK-1 | 2.71 | 3.69 | 36 | 4.01 | 5.82 | 45 |
| g/plant | ZK-2 | 2.49 | 3.37 | 36 | 3.23 | 4.95 | 53 |
|  | ZK-3 | 2.33 | 3.55 | 52 | 3.58 | 5.00 | 40 |
|  | HD | 2.19 | 2.67 | 22 | 3.52 | 5.15 | 47 |
| Protein | ZK-1 | 5.51 | 7.55 | 37 | 7.02 | 9.02 | 29 |
| g/plant | ZK-2 | 5.47 | 7.70 | 41 | 7.08 | 9.36 | 32 |
|  | ZK-3 | 5.12 | 7.20 | 41 | 7.91 | 9.32 | 18 |
|  | HD | 4.06 | 4.90 | 21 | 6.78 | 8.50 | 25 |
